# Interleukin-4 Modulates Neuroinflammation by Inducing Phenotypic Transformation of Microglia Following Subarachnoid Hemorrhage

**DOI:** 10.1007/s10753-023-01917-z

**Published:** 2023-10-29

**Authors:** Jing Wang, Lili Wang, Qingjian Wu, Yichen Cai, Chengfu Cui, Ming Yang, Baoliang Sun, Leilei Mao, Yuan Wang

**Affiliations:** 1 Medical College of Qingdao University, Qingdao, Shandong 266021 China; 2 Institute for Neurological Research, School of Basic Medical Sciences of Shandong First Medical University & Shandong Academy of Medical Sciences, The Second Affiliated Hospital, Taian, Shandong 271000 China; 3 Department of Emergency, Jining No. 1 People’s Hospital, No. 6, Jiankang Road, Jining, Shandong Province 272011 China; 4 Cheeloo College of Medicine, Shandong University, Jinan, 250100 Shandong China; 5 Department of Ultrasonic Diagnosis and Treatment, Shandong Provincial Hospital Affiliated to Shandong First Medical University, Jinan, Shandong 250021 China; 6 Department of Neurology, Shandong Provincial Hospital Affiliated to Shandong First Medical University, Jinan, Shandong 250021 China

**Keywords:** subarachnoid hemorrhage, interleukin-4, microglia, neuroinflammation, microglial activation

## Abstract

Neuroinflammation, a key pathological feature following subarachnoid hemorrhage (SAH), can be therapeutically targeted by inhibiting microglia M1 polarization and promoting phenotypic transformation to M2 microglia. Interleukin-4 (IL-4) is a pleiotropic cytokine known to its regulation of physiological functions of the central nervous system (CNS) and mediate neuroinflammatory processes. However, its specific role in neuroinflammation and microglia responses following SAH remains unexplored. In this investigation, we established both *in vivo* and *in vitro* SAH models and employed a comprehensive array of assessments, including ELISA, neurofunctional profiling, immunofluorescence staining, qRT-PCR, determination of phagocytic capacity, and RNA-Seq analyses. The findings demonstrate an elevated expression of IL-4 within cerebrospinal fluid (CSF) subsequent to SAH. Furthermore, exogenous administration of IL-4 ameliorates post-SAH neurofunctional deficits, attenuates cellular apoptosis, fosters M2 microglia phenotype conversion, and mitigates neuroinflammatory responses. The RNA-Seq analysis signifies that IL-4 governs the modulation of neuroinflammation in microglia within an *in vitro* SAH model through intricate cascades of signaling pathways, encompassing interactions between cytokines and cytokine receptors. These discoveries not only augment comprehension of the neuropathogenesis associated with post-SAH neuroinflammation but also present novel therapeutic targets for the management thereof.

**Graphical Abstract:**

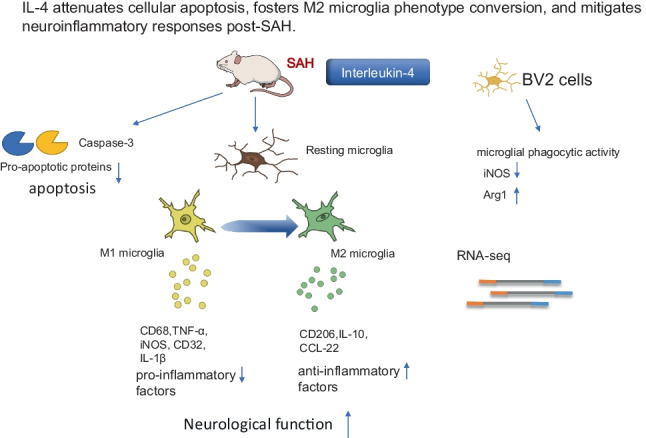

## INTRODUCTION

Subarachnoid hemorrhage (SAH), primarily arising from intracranial arterial rupture, is a catastrophic ailment with significant mortality rates, constituting 5% of all stroke cases [1]. Notwithstanding ongoing enhancements in aneurysm management and neuroimaging methodologies, there has been limited alteration in both mortality and morbidity rates, with a substantial 50% of survivors enduring irreversible impairments [2–4]. The management of SAH and the attainment of favorable functional outcomes persist as formidable challenges.

Neuroinflammation stands as a primary pathological hallmark subsequent to SAH. Following SAH, peripheral immune cells are recruited and activated within damaged tissues, while resident microglia/macrophages undergo activation to initiate the inflammatory cascade [5, 6]. The phenotypes of microglia (M1 and M2 phenotypes) undergo alterations in response to changes in microenvironmental signals, thereby assuming distinct roles [7]. The activated M1 phenotype stimulates elevated levels of pro-inflammatory factors and augments the release of cytotoxic reactive oxygen species (ROS), leading to tissue inflammation and cerebral damage [8]. Conversely, the alternative activated M2 phenotypes release anti-inflammatory cytokines and neurotrophic factors that exert anti-inflammatory effects [9]. Inhibiting microglia M1 polarization while promoting the phenotypic transformation of M2 microglia has proven effective in mitigating acute brain injury, enhancing clinical outcomes [8], and represents a potential therapeutic avenue following SAH.

Interleukin-4 (IL-4), a pleiotropic cytokine, assumes a vital role in modulating the physiological functions of the central nervous system (CNS) and mediating neuroinflammatory processes [10, 11]. The conventional viewpoint suggests that IL-4 is primarily secreted by T helper-like 2 cells. However, emerging research has revealed additional sources, including neurons within ischemic brain tissue and microglial within the CNS themselves, which also contribute to the endogenous defense mechanisms [12, 13]. IL-4 exhibits the ability to modulate immune responses and neuroinflammation, thereby exerting neuroprotective and neurorepair effects in various experimental models of CNS diseases, such as traumatic brain injury, cerebrovascular accident, spinal cord injury, and autoimmune encephalomyelitis [14]. Notably, IL-4 induces a polarized microglia/macrophage phenotype that enhances the clearance of tissues through phagocytosis. Studies have demonstrated that the presence of IL-4, including its exogenous administration, induces the expression of genes characteristic of the M2 microglia phenotype in a focal experimental cerebral ischemia model, subsequently promoting the improvement of neurological function [12]. Early elevation of IL-4 levels in the cerebrospinal fluid of patients with SAH has been found in clinical studies, and IL-4 has been considered to be a protective factor associated with survival [15–17]. However, despite its prominent role in regulating inflammatory responses in other CNS disorders, the effects of IL-4 on microglia following SAH have not been investigated.

The present study aimed to examine the role of IL-4 in regulating neuroinflammation and improving neurological outcomes in both *in vitro* and *in vivo* models of SAH. Through modulation of microglial cell polarization, IL-4 demonstrated its potential in regulating inflammatory responses. We further validated the potential mechanism of IL-4 to modulate inflammatory responses in microglia *in vitro* by RNA-Seq analysis, providing novel therapeutic targets for the treatment of neuroinflammation after SAH.

## MATERIALS AND METHODS

### Animals and SAH Model

A total of 132 adult Sprague–Dawley (SD) rats (male, 260–300 g, 7–8 weeks old) were used in this study. All experimental procedures were reviewed and approved by the Institutional Animal Care and Use Committee of Shandong First Medical University and conformed to the protocols set forth by the National Institutes of Health in the USA (approval No. 2019011).

The SAH model was established using the intravascular puncture technique, as previously described [18, 19]. To summarize, rats were anesthetized with isoflurane (5% for induction and 2.5% for maintenance). The right common carotid artery (CCA) was surgically exposed and the internal carotid artery (ICA) and external carotid artery (ECA) were then isolated from their bifurcations. A nylon wire was inserted into the ICA *via* the ECA and advanced approximately 21–22 mm from the arterial bifurcation until a distinct sensation of penetration persisted for 15 s. In the sham-operated group, the same procedure was followed, excluding the puncturing of the vessel.

### Neurological Function Assessment

Neurologic function was evaluated blindly at 1, 3, and 5 days post-SAH employing a modified Garcia score encompassing six dimensions including spontaneous activity, spontaneous movement of all limbs, forelimb movement, climbing wall of wire cage, reaction to touch on both side of trunk, and response to vibrissae touch. Each dimension was scored on a scale of 0–3 or 1–3, with a minimum total score of 3 and a maximum of 18 [18].

At 5 days after the procedure, the severity of SAH bleeding was evaluated [18]. Briefly, the basal cistern of the rat cranial base was divided into six segments, and each segment was rated on a grade of 0–3 based on the amount of bleeding. The sum of the six regions was the SAH score (0–18), and a score of 0–7 was excluded from the study.

Additionally, the hang wire test was employed to evaluate somatosensory motor function [20]. Briefly, rats were placed on a strip of wire with supports at both ends, and their behavior on the wire within 30 s was recorded for scoring: 0, falling; 1, two front paws hanging from the wire strip; 2, front paws hanging from the wire and attempting to climb up; 3, two front paws and one or two hind paws hanging from the wire strip; 4, four paws hanging from the wire strip with the tail wrapped around the wire strip; and 5, escaping to the supports. The experiment was repeated three times and the average score was taken for calculation.

### Experimental Design *In Vivo*

**Fig. 1:**
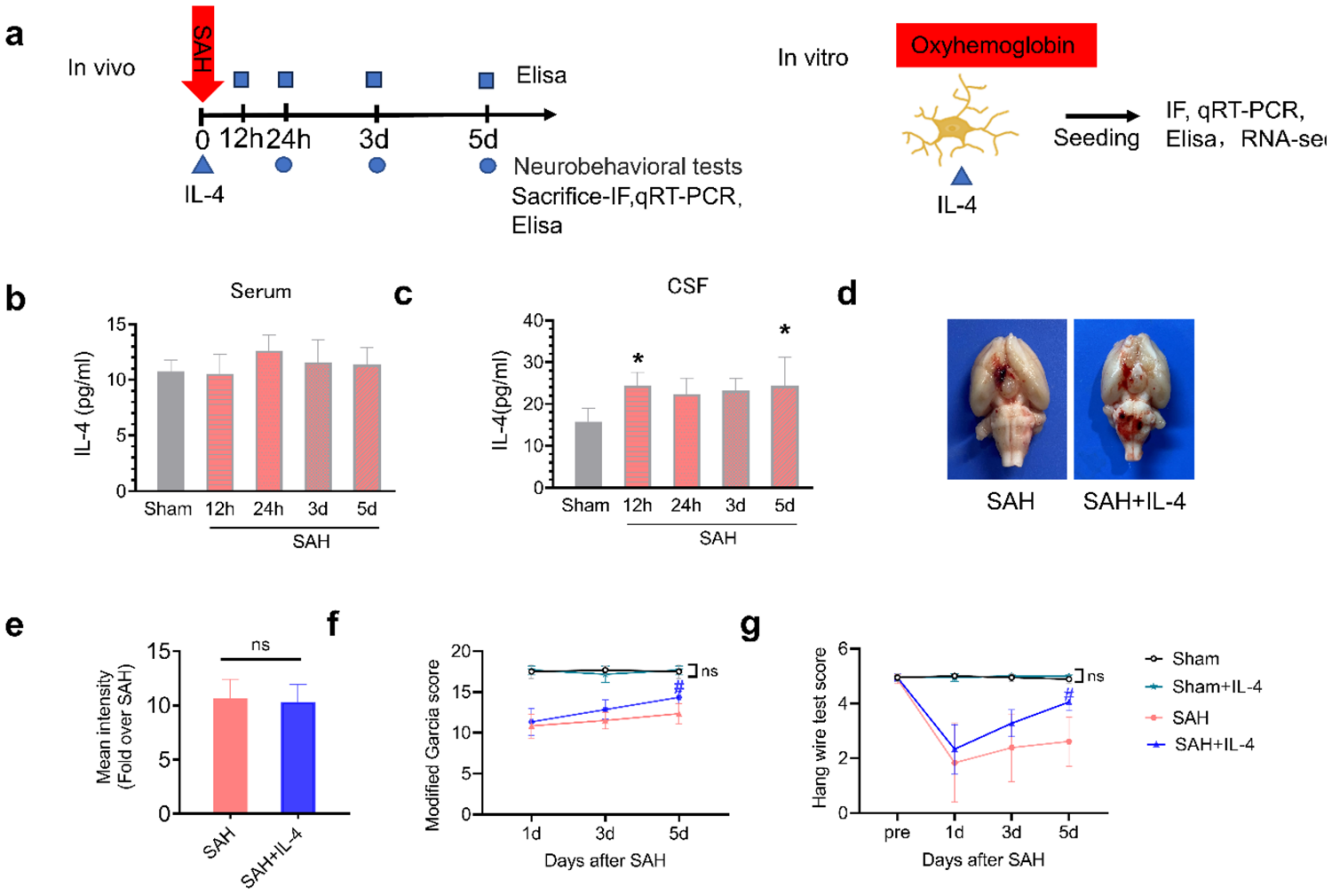
IL-4 levels in peripheral blood and CSF after SAH, and the effects of IL-4 administration on neurofunction. **a** Diagrammatic depiction of the experimental design. **b**–**c** Levels of IL-4 in serum and CSF at 12 h, 1, 3, and 5 days post-SAH in sham group and SAH group (*n* = 5–6 rats/group). **d** Representative images of brain tissues from different groups at 5 days post-SAH. **e** SAH grading scores of brain tissues in different groups at 5 days post-SAH. **f** Modified Garcia score of each group. **g** Hang wire test scores (*n* = 6 rats/group). The data are presented as mean ± SD. ns = not significant, *p < 0.05 vs. sham group, #*p* < 0.05 vs. SAH group.

The experimental design employed is depicted in Fig. 1a. In the *in vivo* experiments, aimed at investigating the alterations in IL-4 expression levels subsequent to SAH, rats were randomly assigned to two groups: the sham group (*n* = 6) and the SAH group (*n* = 36). Peripheral blood and CSF samples were taken for enzyme-linked immunosorbent assay (ELISA) at 12 h, 24 h, 3 days, and 5 days post-SAH.

To examine the impact of IL-4 on neuroinflammation after SAH, rats were randomly divided into four groups: the sham group (administered with phosphate-buffered saline (PBS), *n* = 19), the sham + IL-4 group (administered with IL-4. *n* = 14), the SAH group (subjected to SAH and administered with PBS, *n* = 29), and the SAH + IL-4 group (subjected to SAH and administered with IL-4, *n* = 28). All rats were euthanized 5 days post-SAH, and subsequent analyses included immunofluorescence staining, ELISA, and quantitative real-time polymerase chain reaction (qRT-PCR).

### Enzyme-Linked Immunosorbent Assay

To investigate the alterations in endogenous IL-4 levels following SAH, sham and SAH rats were anesthetized with isoflurane and placed on a stereotaxic apparatus, and CSF samples were collected at the site of the occipital pool [21]. Blood samples were taken by cardiac puncture and then centrifuged for serum (3000 × g for 10 min at 4 °C). IL-4 levels in the CSF and serum samples were assessed using a rat IL-4 ELISA kit, following the manufacturer’s instructions (Mlbio, China).

To assess the impact of IL-4 treatment on the expression levels of inflammatory factors TNF-α, iNOS, and IL-10, brain tissues were obtained from the ipsilateral basal cortex 5 days after SAH and assayed using rat ELISA kits (Mlbio, China). BV2 cell culture supernatants in each group were collected to measure the expression levels of TNF-α, iNOS, and IL-10 through mouse ELISA kits (Mlbio, China). The absorbance density (OD) values were measured at a specific wavelength of 450 nm using a spectrophotometer, and the concentrations of each sample were calculated accordingly.

### Administration of IL-4

IL-4 administration was performed as shown previously [22]. After isoflurane anesthesia, animals were placed on a brain stereotaxic instrument and immobilized. According to the *in vivo* experimental design, an implantable slow-release pump (RWD Life Science, China) containing recombinant rat IL-4 (Peprotech, 60 ng/day) or PBS was positioned into the lateral ventricle of the lesion (coordinates: AP 1.0 mm, ML 1.5 mm, DV 4.5 mm) immediately after the onset of SAH. The pump was then started and the infusion was continued at a constant rate of 0.5 μl/h for 5 days until the rats were euthanized [22].

### Immunofluorescence

Immunostaining was conducted following established protocols [23]. Briefly, brain tissues were collected after cardiac perfusion with cold 4% paraformaldehyde (PFA), placed in 4% PFA overnight, and then dehydrated using 30% sucrose/PBS for 4 days. Tissues at distances of − 2.5 to − 5 mm from bregma were selected for serial sections of 25-μm thickness. Sections were incubated overnight with primary antibody, including anti-NeuN (1:200, MAB377, Abcam), anti-cleaved caspase-3 (1:400, #9661, CST), anti-Iba-1 (1:1000, 019–19741, Wako, Japan), anti-Iba-1 (1:500, ab5076, Abcam), anti-CD68 (1:1000, ab125212, Abcam), and anti-CD206 (1:500, AF2535, R&D, USA), and then incubated for 2 h at room temperature with the appropriate fluorescent secondary antibody (Jackson ImmunoResearch Laboratories). DAPI (Southern Biotech) was utilized for nuclear staining and mounting. Micrographs were taken using a confocal microscope (Olympus, Japan). Three sections per rat were used, and three random fields in the ipsilateral basal cortex were acquired in each section, and the immunopositive cells in the basal cortex were quantified using Image J software [24].

### Cell Culture and Treatment

In the *in vitro* experiments, BV2 cells were divided into three groups for cytophagocytosis assay, qRT-PCR, and RNA-Seq analysis: (1) control group, (2) control +IL-4 group, (3) oxyhemoglobin group, and (4) oxyhemoglobin +IL-4 group. The microglial BV2 cells (purchased from BULEFBIO, China) were cultured in high-glucose DMEM (GIBCO, USA) media supplemented with 10% fetal bovine serum (FBS) and 1% penicillin–streptomycin, and incubated in a humidified environment at 37 °C with 5% CO2. Oxyhemoglobin (10 μM, Shanghai Yuanye Bio-Technology Co., China) was added to the medium for 24 h to induce *in vitro* SAH [25]. For the control +IL-4 group and oxyhemoglobin + IL-4 group, 20 ng/ml IL-4 was administered for 24 h of treatment with reference to previous descriptions before [26] proceeding to the next step of the study. DMSO was used as a control for the treatment conditions.

### Phagocytosis Assay

As described above, BV2 cells were inoculated into 24-well plates and incubated for 24 h, and then the *in vitro* SAH model was induced, followed by 24 h of incubation with or without IL-4. Fluorescent microbeads with a diameter of 1 μm (Invitrogen, diluted 1:15,000) were introduced into the culture medium and incubated with the cells for 4 h. The BV2 cells were then fixed on cover slips using 4% paraformaldehyde. Immunofluorescent staining with phalloidin was performed to label the cell cytoskeleton, while DAPI was used to stain the cell nuclei for 5 min. Laser confocal microscopy was used for imaging, and Image J software was used to count the number of microglia phagocytosed microspheres in different groups by randomly selecting 3 fields of view within 4 wells in each group [27, 28].

### qRT-PCR

Total RNA was extracted from rat brain basal cortex tissue and BV2 cells using the Total RNA Kit (Qiagen, Santa Clara, CA). The isolated RNA was then reverse transcribed into complementary DNA (cDNA) through the reverse transcription process, employing the First Strand cDNA Synthesis Kit (Yeasen, Shanghai, China) according to the manufacturer’s instructions. Amplification was subsequently performed as per the manufacturer’s protocol. The endogenous reference gene primers for GAPDH in rats and mice, as well as other qRT-PCR primers, were obtained from Sangon Technology (Shanghai, China) and are listed in Table 1. All experiments were repeated three times. The relative expression levels were calculated using the 2 − ΔΔCt method, with GAPDH mRNA serving as an internal control for normalization.

**Table 1:** Primers Used in the qRT-PCR Reaction

	Gene	Forward primer (5′-3′)	Reverse primer (5′-3′)
For rat experiments	Tnf α	CCCAGACCCTCACACTCAGATCAT	CAGCCTTGTCCCTTGAAGAGAA
	iNOS	CAAGCACCTTGGAAGAGGAG	AAGGCCAAACACAGCATACC
	CD32	AATCCTGCCGTTCCTACTGATC	GTGTCACCGTGTCTTCCTTGAG
	IL-1β	TCTCACAGCAGCATCTCGACAAG	CCACGGGCAAGACATAGGTAGC
	IL-10	AAGGCAGTGGAGCAGGTGAAG	CACGTAGGCTTCTATGCAGTTGATG
	CCL-22	CTGATGCAGGTCCCTATGGT	GCAGGATTTTGAGGTCCAGA
For BV2 cell experiments	iNOS	CAAGCACCTTGGAAGAGGAG	AAGGCCAAACACAGCATACC
	Arg1	TCACCTGAGCTTTGATGTCG	CTGAAAGGAGCCCTGTCTTG
	Aqp1	TTGACTACACTGGCTGCGGTATC	GTTTGAGAAGTTGCGGGTGAGC
	Mgl2	CTAACAGTTCCTTCCCAGTCCTTCC	CACGGAGATGACCACCAGTAGC
	Mmp13	CTTCCTGATGATGACGTTCAAG	GTCACACTTCTCTGGTGTTTTG
	Cybb	GACAGGAACCTCACTTTCCATA	TGAAGAGATGTGCAATTGTGTG
	Mmp12	TGTACAGCATCTTAGAGCAGTG	TATGTAGTCTACATCCTCACGC
	Cx3cr1	TCGGTCTGGTGGGAAATCTGTTG	CAGGTTCAGGAGGTAGATGTCAGTG
	Ccl9	CTGCCCTCTCCTTCCTCATTCTTAC	TGCTGTGCCTTCAGACTGCTC

### RNA Sequencing (RNA-Seq) Analysis

Total RNA was isolated from BV2 cells of both the oxyhemoglobin group and oxyhemoglobin + IL-4 group, with subsequent sequencing analysis primarily carried out by BGI Corporation (China) [29]. Differential expression analysis was carried out using DESeq2 with a log-fold change (FC) threshold of > 0.5 and *q* value (*p*-adjusted) < 0.05 to identify differentially expressed genes (DEGs). Gene ontology (GO) analysis, KEGG pathway analysis, and protein–protein interaction (PPI) network analysis were all performed on the Dr. Tom network platform of BGI (online analysis and visualization website: http://report.bgi.com), and *q* value < 0.05 was considered pathway enrichment. The protein–protein interaction (PPI) was mapped by String (https://string-db.org/).

### Statistical Analysis of Data

All data in this study were presented as mean ± standard deviation (SD) and analyzed using GraphPad Prism 9.0 software (USA). Unpaired Student *t*-test was used to compare the two groups, while one-way analysis of variance (ANOVA) was used between multiple groups followed by Tukey’s post hoc tests. The Mann–Whitney test (for two groups) or the Kruskal–Wallis test followed by Dunn’s post hoc test (for multiple groups) was employed to examine variables that did not conform to a normal distribution. Two-way ANOVA with Bonferroni post hoc test was performed for modified Garcia score and hang wire test. *p* < 0.05 was considered statistically significant.

## RESULTS

### The Expression Level of IL-4 in CSF Was Increased After SAH

In this study, we measured the expression levels of IL-4 in peripheral blood serum and CSF of rats at 12 h, 24 h, 3 days, and 5days after SAH using ELISA. The results showed that compared to the sham group rats, there was no significant change in IL-4 expression levels in the serum at all time points after SAH (Fig. 1b, *p* > 0.05). However, we observed a significant increase in IL-4 expression in the CSF of rats at 12 h and 5 days after SAH (Fig. 1c, *p* < 0.05).

### IL-4 Administration Improves Neurological Deficits Following SAH

Furthermore, we examined the impact of IL-4 administration on neurological deficits after SAH. The endovascular perforation model was utilized to induce SAH in rats, and on the 5th day post-surgery, visible blood clots were observed in the Willis circle of all SAH groups, with no statistically significant differences in SAH grading scores among the different SAH groups (Fig. 1d–e, *p* > 0.05). To assess the potential improvement in sensory-motor function after SAH, modified Garcia tests and hang wire tests were performed at 1, 3, and 5 days post-SAH. The results showed that there was no statistically significant difference in neurological function scores between the sham group and the sham + IL-4 group at each observed time point (*p* > 0.05). Compared to the sham group, the SAH group exhibited significant neurological functional impairments, which gradually recovered over time. However, treatment with IL-4 facilitated this functional recovery, as indicated by the modified Garcia test and hang wire test scores at 5 days (Fig. 1f, g, *p* < 0.05). These findings suggest that IL-4 administration can improve neurological deficits after SAH.

### IL-4 Administration Attenuates Neuronal Apoptosis After SAH

**Fig. 2:**
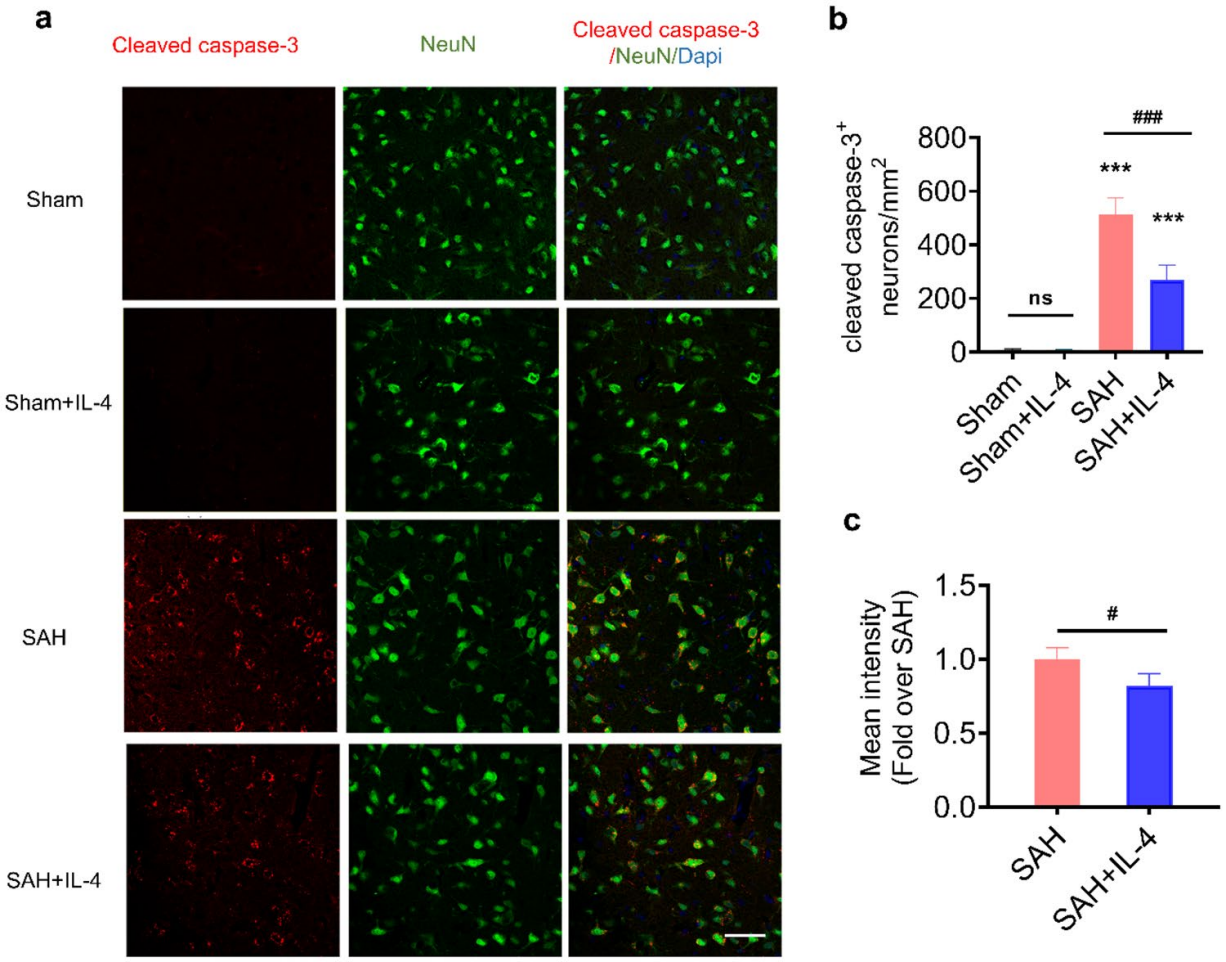
IL-4 delivery attenuates cell apoptosis in rats post-SAH. **a** Representative immunofluorescence images of the basal cortex at 5 days post-SAH in rats. **b** Quantification of cleaved caspase-3^+^ neurons. **c** Quantification of the fluorescence density value of cleaved caspase-3. ns = not significant, ***p < 0.001 vs. sham group, #*p* < 0.5, ###*p* < 0.001, *n* = 4 animals/group, scale bar = 50 μm.

Subsequently, we assessed the potential of continuous ventricular delivery of IL-4 over a 5-day period to rescue neuronal damage following SAH. Immunofluorescent staining targeting the apoptosis-related factor cleaved caspase-3 and the neuronal marker NeuN was performed in the basal cortex of the brain post-SAH (Fig. 2). The results depicted in Fig. 2b demonstrate a significant increase in the number of cleaved caspase-3-positive neurons in the basal cortex of the SAH group compared to the sham group after 5 days, whereas IL-4 treatment significantly decreased this number (*p* < 0.05). There was no significant difference between sham + IL-4 group and sham group (*p* > 0.05). Furthermore, the immunofluorescence intensity of cleaved caspase-3 was quantified, revealing a marked reduction in its levels in the basal cortex after SAH with IL-4 treatment (Fig. 2c, *p* < 0.05). These findings provide evidence that IL-4 administration can ameliorate cellular apoptosis in rats induced with SAH.

### IL-4 Promotes Microglial Phenotype Switch and Alleviates Neuroinflammation After SAH

**Fig. 3:**
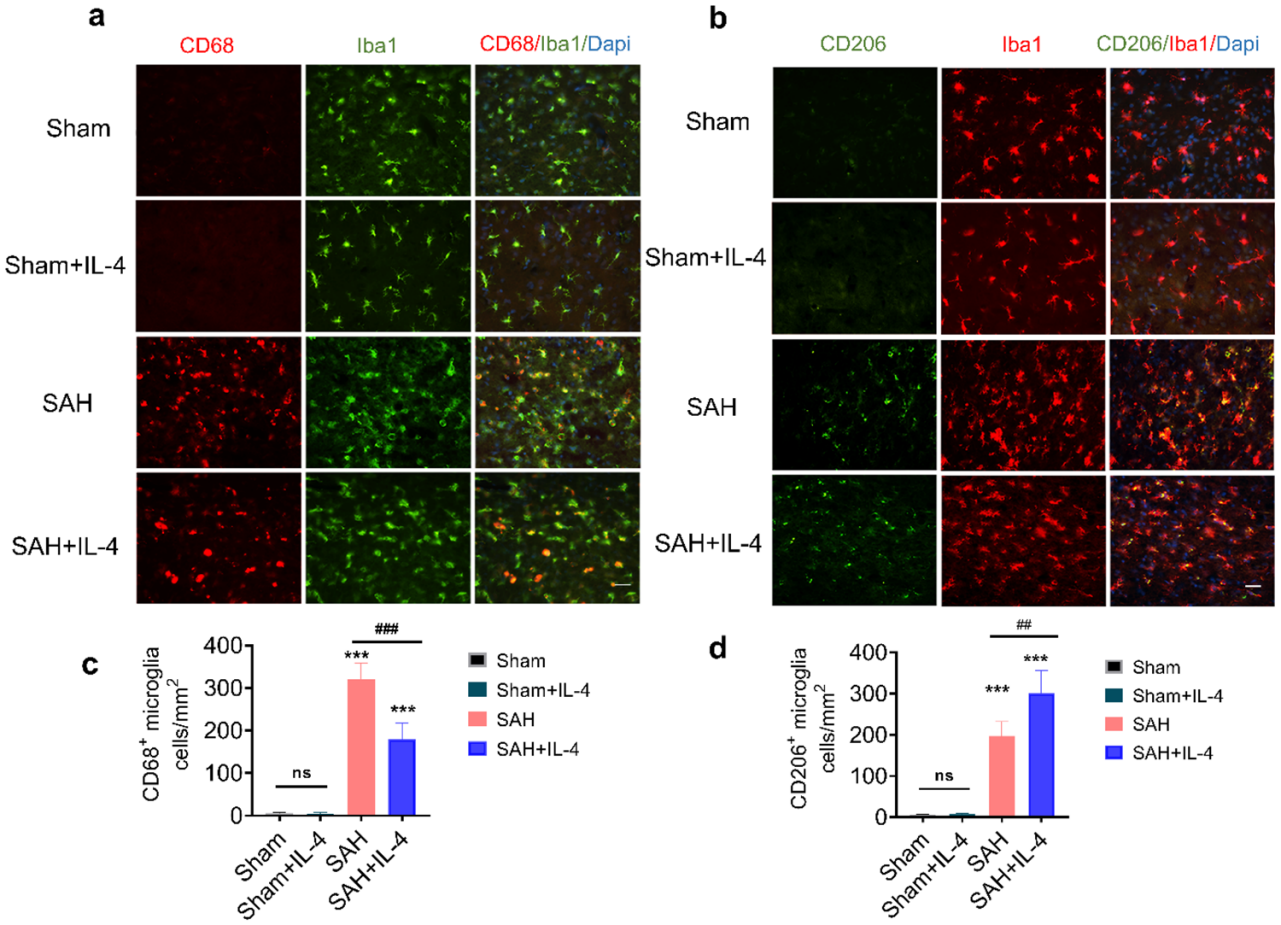
IL-4 regulates microglial polarization post-SAH. **a**–**b** Representative immunofluorescence images of CD68/Iba1 and CD206/Iba1 in the basal cortex at 5 days post-SAH. **c**–**d** Quantification of CD68^+^/Iba1^+^ cells and CD206^+^/Iba1^+^ cells. ns = not significant, ***p < 0.001 vs. sham group, ##*p* < 0.1, *n* = 4 animals/group, scale bar = 20 μm.

To investigate the effects of IL-4 on microglial polarization after SAH, we conducted double immunostaining of Iba1 with M1 phenotype marker CD68 and M2 phenotype marker CD206 in the basal cortex. Our results demonstrated a significant increase in the number of CD68-positive Iba1 cells, indicating an activation of M1 microglia, after SAH compared to the sham group. However, treatment with IL-4 significantly reduced the co-localization of CD68-positive Iba1 cells (Fig. 3a, c, *p* < 0.05). Furthermore, we examined the expression of M2 microglia and found a significant increase in CD206-positive Iba1 cells after IL-4 treatment compared to the SAH group (Fig. 3b, d, *p* < 0.05). Notably, IL-4 treatment did not alter the activation of microglia in sham rats. These findings suggest that IL-4 has a positive impact on microglial phenotype switch and can alleviate neuroinflammation after SAH.

**Fig. 4:**
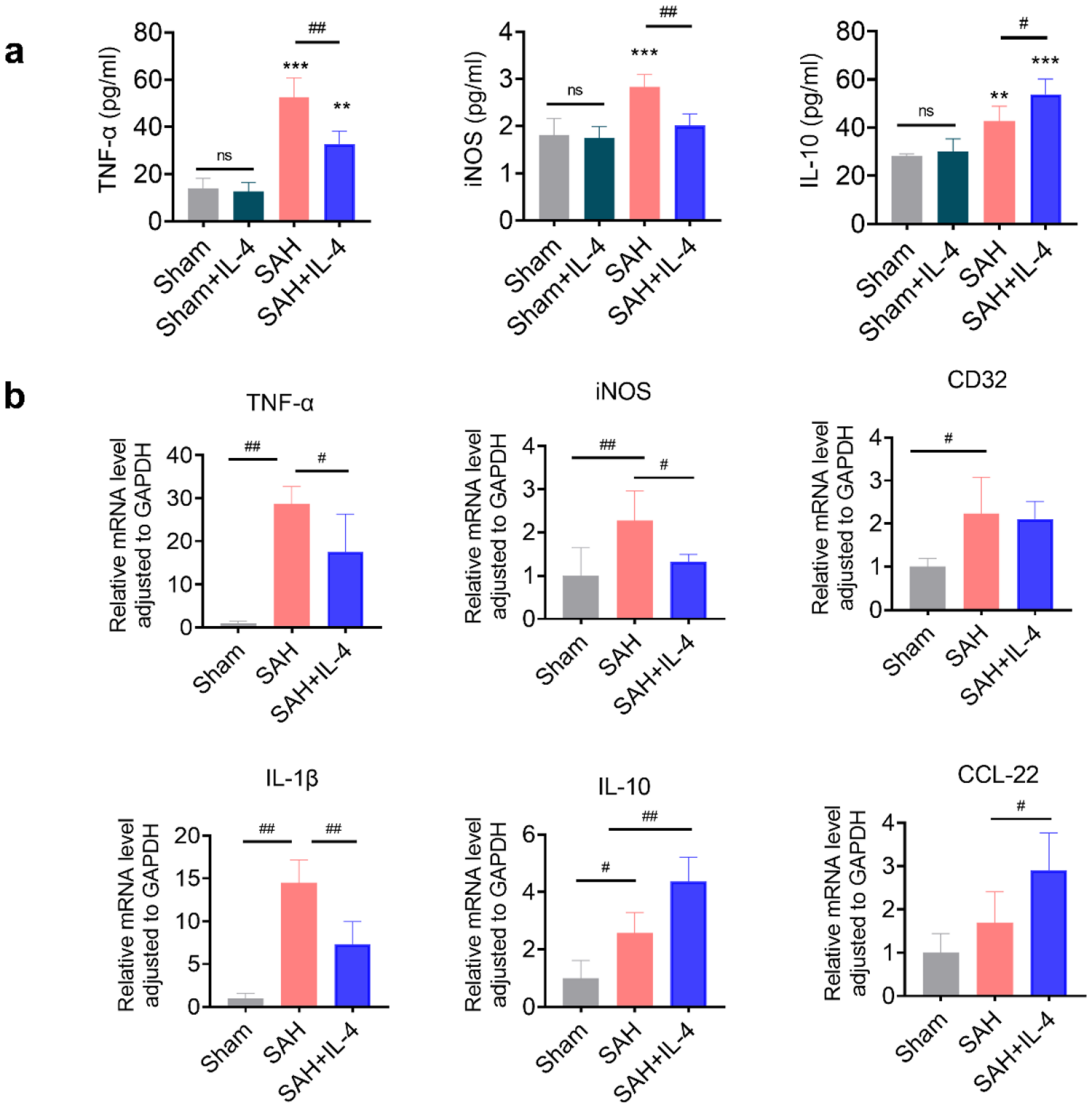
IL-4 regulates the levels of inflammation-associated factors after SAH. **a** The quantification of TNF-α, iNOS, and IL-10 expression levels through ELISA at 5 days after SAH. *n* = 4 rats/group. **b** The qRT-PCR analysis of pro-inflammatory mediators TNF-α, iNOS, CD32, IL-1β, and anti-inflammatory factors IL-10 and CCL-22 in rats at 5 days post-SAH. ns = not significant, **p < 0.01,***p < 0.001 vs. sham group, #*p* < 0.05, ##*p* < 0.1, *n* = 5 rats/group.

We conducted ELISA to assess the expression levels of inflammatory factors TNF-α, iNOS, and IL-10 in the ipsilateral basal cortex of rat brain tissue after SAH (Fig. 4a). The results showed that IL-4 treatment did not significantly alter the expression levels of TNF-α, iNOS, and IL-10 in the sham group (*p* > 0.5). However, SAH induction increased the expression levels of TNF-α, iNOS, and IL-10. Notably, IL-4 treatment significantly reduced the expression levels of pro-inflammatory cytokines TNF-α and iNOS, while simultaneously increasing the level of the anti-inflammatory cytokine IL-10.

We further validated the expression of six cytokines associated with neuroinflammation in the sham group, SAH group, and SAH + IL-4 group using qRT-PCR. Five days after SAH, compared to the sham group, the expression of pro-inflammatory markers (including TNF-α, iNOS, CD32, IL-1β) in the brains of SAH rats treated with the vehicle was significantly increased. However, after 5 days of IL-4 treatment, the expression of TNF-α, iNOS, and IL-1β was significantly suppressed. Additionally, the anti-inflammatory cytokine IL-10 was observed to be upregulated following the occurrence of SAH, and both CCL-22 and IL-10 exhibited a substantial increase after a 5-day administration of IL-4 (Fig. 4b, *p* < 0.05). These findings suggest that IL-4 treatment possesses the capacity to regulate the neuroinflammatory response subsequent to SAH.

### IL-4 Enhances the Phagocytic Efficiency of Microglial Cells and Regulates the Inflammatory Response *In Vitro*

**Fig. 5:**
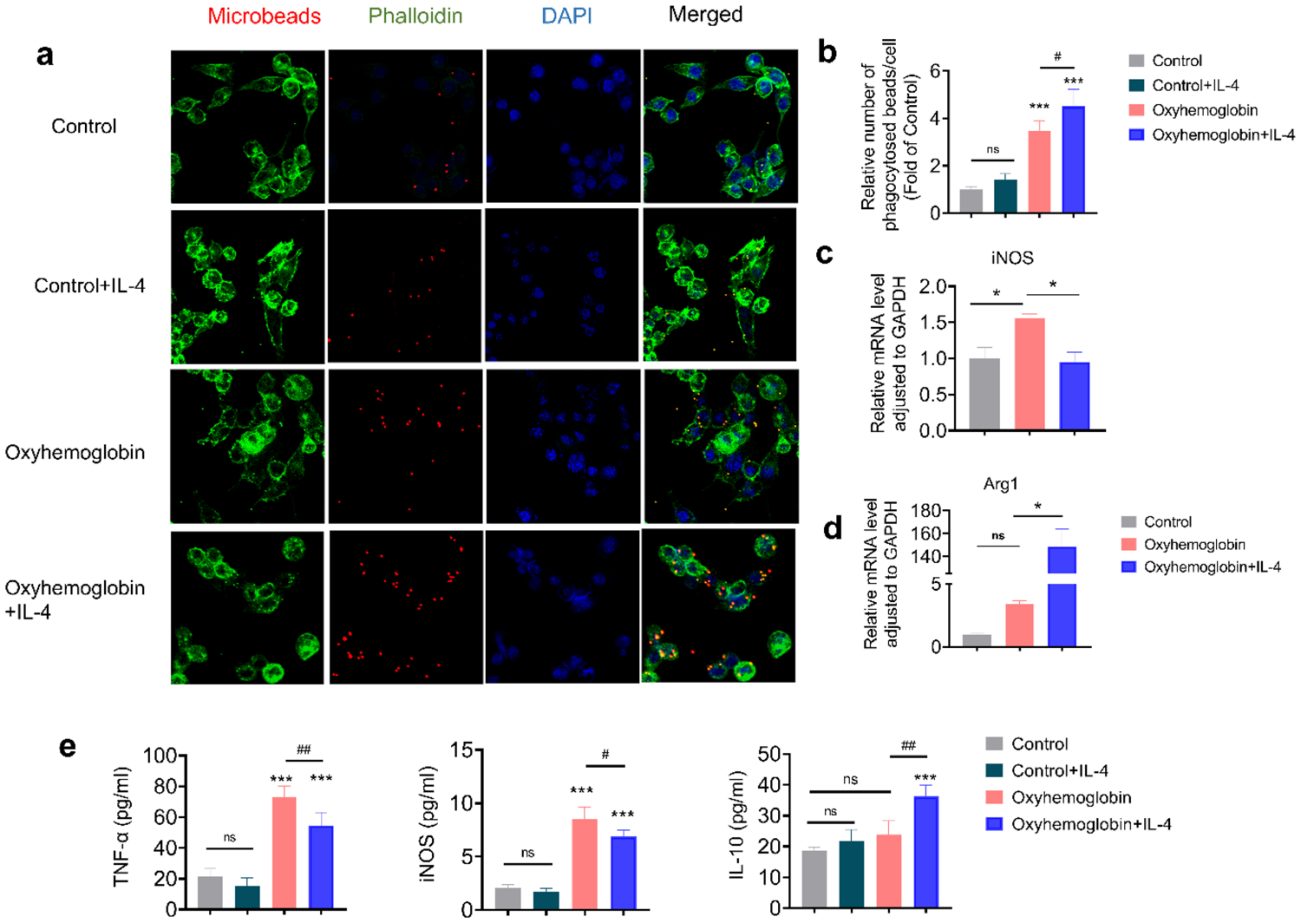
IL-4 promotes microglial phagocytic activity. **a** Evaluation of IL-4’s effect on microglial phagocytosis by assessing the number of fluorescent microbeads (red) within the cells. Phalloidin (green) labels the cellular cytoskeleton, and DAPI (blue) stains the cell nuclei. **b** Quantitative analysis of fluorescent microbeads per cell. *n* = 4. **c**–**d** qRT-PCR analysis of expression levels of pro-inflammatory mediator iNOS and anti-inflammatory factor Arg1 in BV2 cells. **e** The quantification of TNF-α, iNOS, and IL-10 expression levels through ELISA *in vitro*. Results are representative of four independent experiments. Bar = 20 μm. ns = not significant, ***p < 0.001 vs. sham group, #*p* < 0.05, ##*p* < 0.01, ###*p* < 0.01.

The engulfment of cellular debris and deceased or damaged cells represents a pivotal function of microglial cells in the context of inflammatory immune reactions. To gain deeper insights into the association between IL-4 administration and microglial cell regulation, we induced the SAH model *in vitro* and administered IL-4 in the treatment group. Through quantitative analysis utilizing fluorescent microbeads, it was observed that cellular phagocytosis was significantly enhanced in the SAH model *in vitro* (*p* < 0.001), and the phagocytic functionality of the oxyhemoglobin + IL-4 group significantly surpassed that of the oxyhemoglobin group (Fig. 5a–b, *p* < 0.05). IL-4 had no effect on the phagocytosis of BV-2 cells in the control group.

We next assessed the role of IL-4 treatment in inflammatory response in BV2 microglial cells using qRT-PCR and ELISA. The results from ELISA analysis indicated that there was no significant difference in the expression levels of TNF-α, iNOS, and IL-10 between the control + IL-4 group and the control group (Fig. 5e). In the *in vitro* SAH model, we observed a substantial increase in TNF-α and iNOS levels in BV2 cells compared to the control group. However, when IL-4 treatment was administered, the levels of TNF-α and iNOS were significantly reduced. Additionally, IL-4 treatment resulted in a significant increase in the level of the anti-inflammatory cytokine IL-10 after SAH (*p* < 0.05). The results of qRT-PCR were largely consistent with the results of the *in vivo* experiments, in which the *in vitro* SAH model resulted in heightened levels of the M1 pro-inflammatory marker iNOS in microglial cells, whereas the levels of iNOS were significantly reduced after IL-4 administration (Fig. 5c). Concurrently, IL-4 treatment significantly resulted in a significant elevation in the expression levels of the M2 phenotype pro-inflammatory marker Arg-1 (Fig. 5d, *p* < 0.05). These results underline the regulatory role of IL-4 on the phagocytic function and inflammatory response of microglial cells *in vitro*.

### RNA-Seq Analysis of BV2 Cells *In Vitro*

**Fig. 6:**
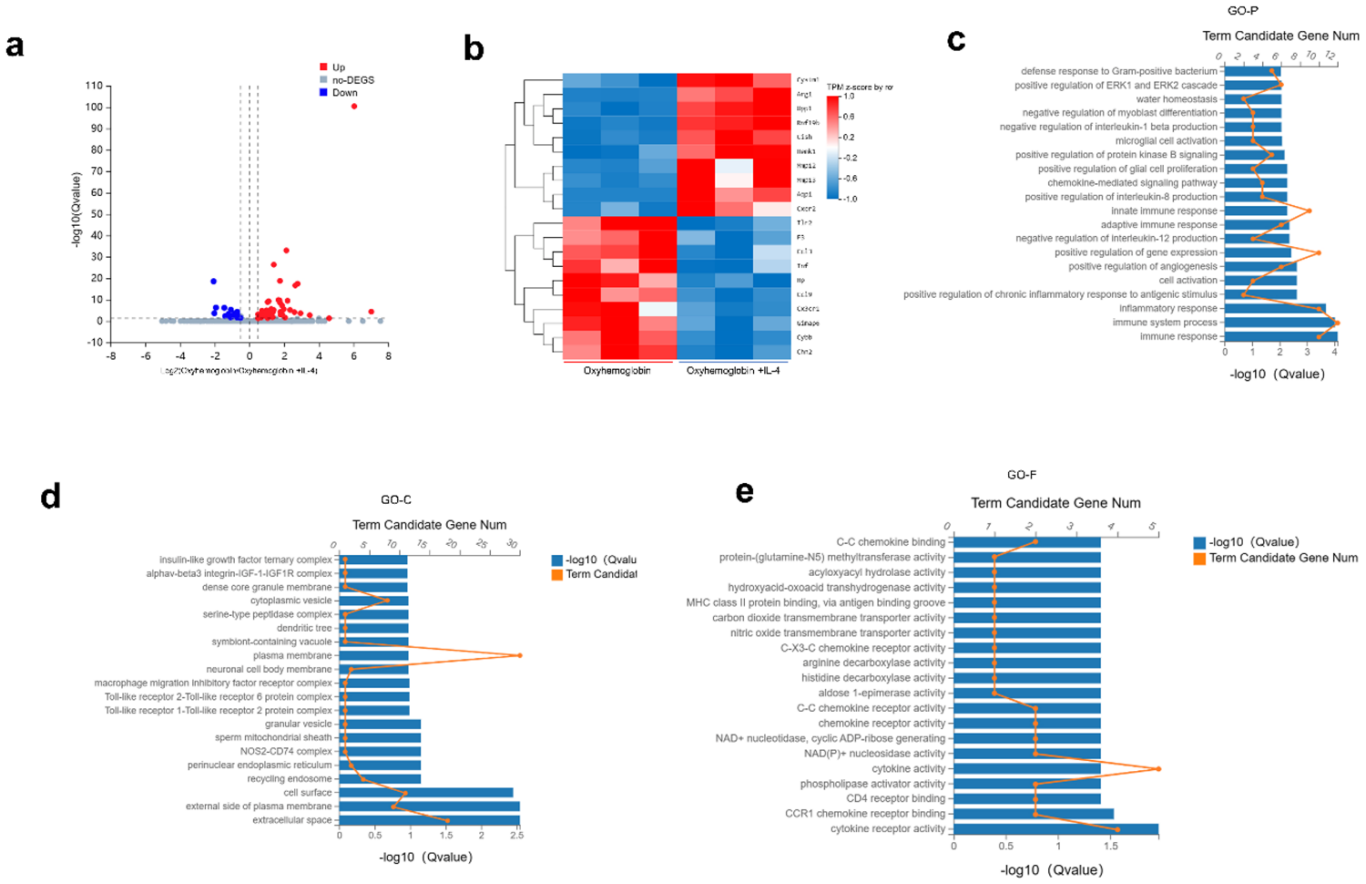
RNA-Seq analysis of different groups of microglial cells. **a**–**b** Volcano plot and heatmap depicting DEGs between the oxyhemoglobin group and oxyhemoglobin + IL4 group. In the volcano plot, upregulated genes are indicated in red, while downregulated genes are indicated in blue. **c**–**e** Further analysis of differentially expressed genes through GO enrichment analysis, including biological processes, cellular components, and molecular functions. Results are representative of 3 independent experiments.

To elucidate the biological processes that may be altered by IL-4 interference, we conducted RNA-Seq analysis on BV2 microglial cells in oxyhemoglobin and oxyhemoglobin + IL-4 groups. We identified a total of 72 differentially expressed genes (DEGs), consisting of 21 upregulated genes and 51 downregulated genes (Fig. 6a), of which 10 upregulated and 10 downregulated genes were depicted in the heatmap (Fig. 6b). Subsequently, we conducted Gene Ontology (GO) enrichment analysis on these 72 commonly regulated DEGs. GO enrichment unveiled significant statistical enrichment of genes involved in diverse biological processes, encompassing immune response, immune system process, inflammatory response, positive regulation of gene expression, and positive regulation of ERK1 and ERK2 cascade (Fig. 6c). In terms of cellular component categorization, the top-enriched cluster encompassed plasma membrane, cell surface, and the external side of the plasma membrane (Fig. 6d). Functional analysis of DEGs enrichment primarily associated with cytokine receptor activity, CCR1 chemokine receptor binding, and CD4 receptor binding (Fig. 6e).

**Fig. 7:**
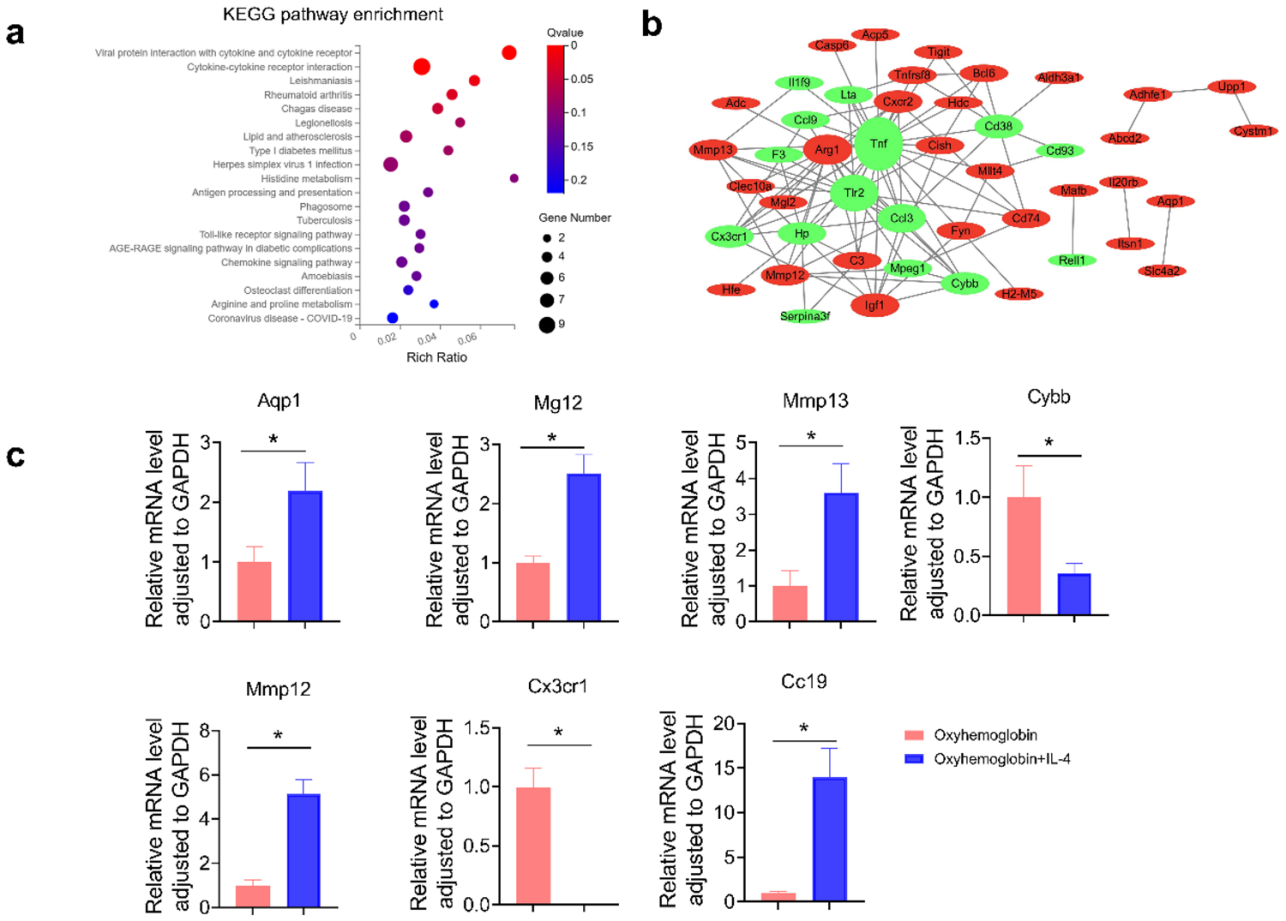
KEGG analysis of DEGs in microglial cells from different groups. **a**–**b** KEGG pathway enrichment and PPI network analysis. Results are representative of three independent experiments. **c** Evaluation of the expression levels of DEGs, including Aqp1, Mg12, Mmp13, Cybb, Mmp12, Cx3cr1, and Ccl9, by qRT-PCR. Results are representative of four independent experiments. *p < 0.05.

Subsequently, we performed KEGG pathway enrichment and PPI analysis of the DEGs. The highest ranked pathway based on the number of genes involved was the cytokine-cytokine receptor interaction pathway (Fig. 7a). In the PPI network, the 10 key genes regulating IL-4 in SAH-induced neuroinflammation were Tnf, Tlr2, Arg1, Ccl3, Cxcr2, Cx3cr1, Cybb, Hp, Cd74, and Ccl9, with Tnf being associated with a variety of biological processes (Fig. 7b). Hence, we hypothesize that Tnf plays a crucial role in the IL-4-mediated modulation of neuroinflammation following SAH. The relative levels of upregulated genes Aqp1, Mg12, Mmp13, Mmp12, and Ccl9 and downregulated genes Cybb and Cx3cr1 were verified by qRT-PCR (Fig. 7c).

## DISCUSSION

IL-4 is a mammalian-specific cytokine that plays a pivotal role as a pleiotropic cytokine in numerous immune/inflammatory pathways [14], particularly in the development of type 2 inflammatory responses and the polarization of macrophages towards an M2 phenotype [30, 31]. As a significant anti-inflammatory, it plays a protective role in brain tissue after ischemic injury [22]. However, its role in the pathophysiology of SAH remains unclear.

Clinical studies in SAH patients have reported elevated [15] or unchanged levels [16] of IL-4 in peripheral blood during the acute phase, whereas the levels of IL-4 in the CSF are elevated and have been considered a protective factor associated with survival [17] and early compensatory anti-inflammatory response [16]. In experimental animal models of SAH, activation of A3 adenosine receptors upregulated IL-4 expression and ameliorated early brain injury following SAH [22]. Therefore, enhancing IL-4 levels holds promising therapeutic potential in promoting neurofunctional recovery after SAH. In our study, we observed no significant change in serum IL-4 expression levels in rats after SAH, while expression levels in the CSF significantly increased at 12 h and 5 days. This increase is believed to be, at least in part, a result of the infiltration and accumulation of T cells and other immune cells in the injured brain.

Neuroinflammation is a major pathological hallmark following SAH. Activation of resident microglia in the CNS and infiltration of macrophages are key events targeted by IL-4 for SAH treatment [20]. While it is well-established that IL-4 acts as a promoter of M2 macrophage polarization [32], the impact of exogenous IL-4 on the polarization state of microglia/macrophages after SAH has not been previously investigated. Here, we explore the functional response of BV2 microglial cells in SAH animal models and *in vitro* culture upon IL-4 administration. In the animal models, IL-4 administration resulted in neurological functional recovery, reduced apoptosis of cortical neurons, phenotypic transition of microglia towards an anti-inflammatory M2 phenotype, and suppression of pro-inflammatory factors including TNF-α, iNOS, and IL-1β. *In vitro* experiments demonstrated that IL-4 enhanced the phagocytic capacity of microglial cells and alleviated neuroinflammation. In the current study, we found that IL-4 treatment did not have an impact on microglia activation in sham rats or the regulation of inflammation and phagocytosis in control BV2 cells, which is consistent with the results of a recent study [33]. It is worth noting that previous studies have mainly focused on the long-term neuroprotective effects of IL-4 [22, 34], whereas our results demonstrate its beneficial effects on neurofunction occurring at 5 days post-SAH. Although longer-term observation was not carried out, our findings suggest significant potential for sustained functional recovery.

RNA-Seq analysis of microglia confirmed that IL-4 regulates various inflammatory pathways, primarily through cytokine-cytokine receptor interaction. Tnf, Tlr2, Arg1, Ccl3, Cxcr2, Cx3cr1, Cybb, Hp, Cd74, and Ccl9 were identified as key driver genes regulated by IL-4 administration *in vitro* SAH models. Among them, Tnf is associated with multiple biological processes including cytokine-cytokine receptor interaction and cell apoptosis. The TNF-α signaling pathway is widely recognized to assume a crucial role in the pathogenesis of SAH, and its signaling process is intricate, regulating SAH through many interconnected pathways. Targeting TNF-α function has the potential to ameliorate SAH-induced brain damage and serve as a therapeutic target [35–37].

It is noteworthy that we discovered an upregulation of the chemokine receptor CXCR2 in microglial cells after IL-4 treatment. A study examining immune-regulatory biomarkers in blood and tissue samples of SAH patients also indicates the involvement of CXCR2 in SAH progression [38]. While it is known that CXCR2 is upregulated after stroke to recruit neutrophils to the brain, blocking CXCR2 does not necessarily improve neurofunctional outcomes [39–41]. Rapid downregulation of CXCR2 may impact neuronal-glial communication [42]. Some researchers have also found that IL-4 incubation upregulates CXCR2 expression in human monocytes and macrophages [43], and the combination of IL-4 and TNF-α induces autocrine secretion of CXCR2 chemokines [44]. However, further investigation is required to clarify the impact of IL-4 on CXCR2 after SAH and its contribution to SAH. Overall, current research suggests that the beneficial effects of IL-4 cannot be attributed solely to the actions of a single cell type but rather to complex cascade reactions.

Despite promising results of IL-4 treatment in animal models of CNS diseases, further research is imperative to determine the most optimal delivery route of administration for patients. Intravenous delivery holds prominence in clinical practice; however, it is essential to note that IL-4 exhibits a markedly abbreviated half-life [45, 46]. In the context of targeting cerebrovascular accidents, as exemplified in our investigation, the administration of IL-4 into the CSF is frequently employed. Nevertheless, it is imperative to acknowledge that the employment of stereotactic surgical techniques in our research is invasive and engenders potential hazards, including infection and surgery-induced secondary brain damage. Consequently, the generalizability of this approach to subarachnoid hemorrhage (SAH) patients may be restricted. Gene engineering approaches have been explored for IL-4 expression [47], but the optimal clinical delivery method remains to be investigated. In addition, the BV2 microglial cell model *in vitro* has helped to elucidate the molecular mechanisms of IL-4 therapy but has not been studied on neurons or astrocytes, which in fact communicate with each other and play multiple roles in the presence of IL-4 [48, 49].

In conclusion, this study demonstrates, for the first time, that exogenous IL-4 administration effectively enhances neurobehavioral performance and mitigates neuronal apoptosis post-SAH, which may be attributed to a protective mechanism that induces M2 phenotypic transformation in microglia/macrophages. IL-4 governs the polarization of microglia and the secretion of pro-inflammatory cytokines by means of complex signaling pathways, including cytokine-cytokine receptor interactions.

## Data Availability

The original datasets generated for this study are available on request to the corresponding author.
